# Crohn’s disease and schistosomiasis: a rare association

**DOI:** 10.11604/pamj.2016.25.124.10718

**Published:** 2016-10-31

**Authors:** Faten Limaiem, Asma Sassi, Sabeh Mzabi

**Affiliations:** 1 University of Tunis El Manar, Faculty of Medicine of Tunis, 1007, Tunisia

**Keywords:** Crohn´s disease, schistosomiasis, intestine, pathology

## Abstract

Schistosomiasis is a chronic enteropathogenic disease caused by blood flukes of the genus Schistosoma. Coexistence of schistosomiasis with Crohn's disease is very rare. To the best of our knowledge, this association has been described in literature only once. A 20-year-old male patient with a past medical history of appendectomy and ileocecal Crohn's disease, presented with abdominal pain and vomiting. Ileocolonoscopy showed an ulcerated and congested appearance of the upper rectum and sigmoid. Computed tomography scan revealed a circumferential thickening of the terminal ileum with luminal stenosis. Histopathological examination of the biopsy specimens revealed a focally ulcerated colonic epithelium. The lamina propria was fibrous harbouring a polymorphic inflammatory infiltrate including lymphocytes and plasma cells organized in lymphoid follicles admixed with eosinophils and neutrophils. In the submucosa, there were two well-preserved schistosoma eggs surrounded by a thick shell with a barely visible terminal spine. The final pathological diagnosis was colonic schistosomiasis associated with Crohn's disease. The patient underwent an ileocecal resection for stenosis of the terminal ileum complicated with enterocutaneous fistula. The postoperative course was uneventful. A stool examination and serology tests were planned for this patient who was lost to follow-up.

## Introduction

Schistosomiasis or bilharziosis is a chronic enteropathogenic disease caused by blood flukes of the genus *Schistosoma* [1]. The association of Crohn's disease with schistosmiasis is exceptional with only one case reported in literature [2]. In this paper, the authors report a new case of Crohn's disease associated with schistosomiasis in a Tunisian patient who traveled to an endemic country.

## Patient and observation

**Figure 1:**
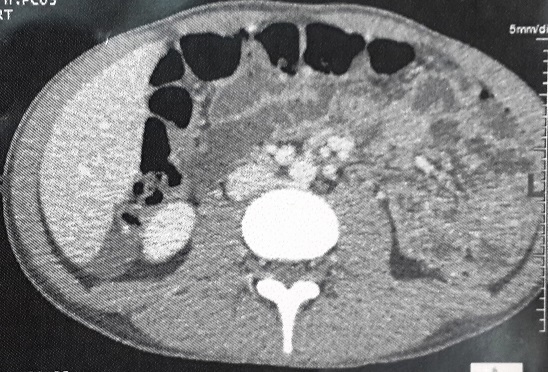
Computed tomography scan revealed a circumferential thickening of the terminal ileum with luminal stenosis and dilated intestine upstream: mesenteric lymphadenopathies were also noted

**Figure 2:**
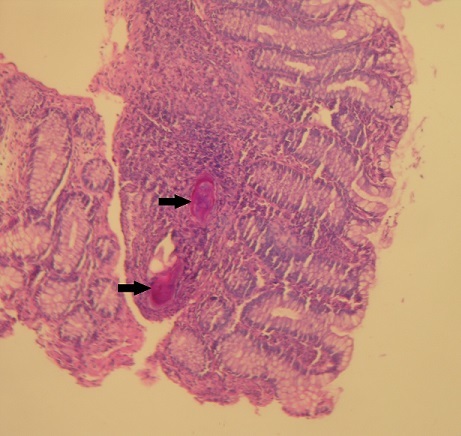
In the submucosa of the colon, there were two ovoidal and pear shaped schistosoma ova (black arrows) (hematoxylin and eosin, magnification × 40)

**Figure 3:**
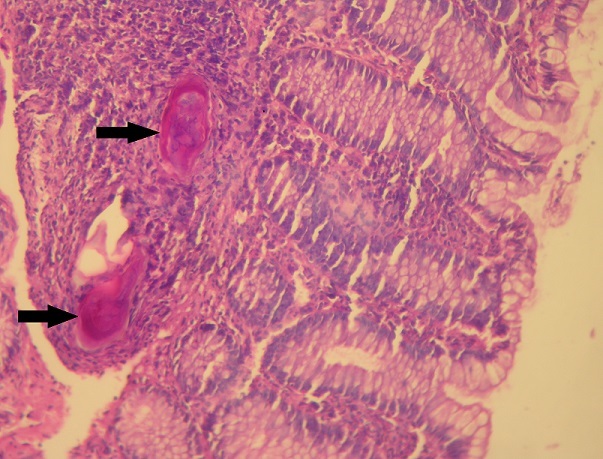
Histological section showing two schistosoma eggs in the colonic submucosa (black arrows) (hematoxylin and eosin, magnification × 200)

**Figure 4:**
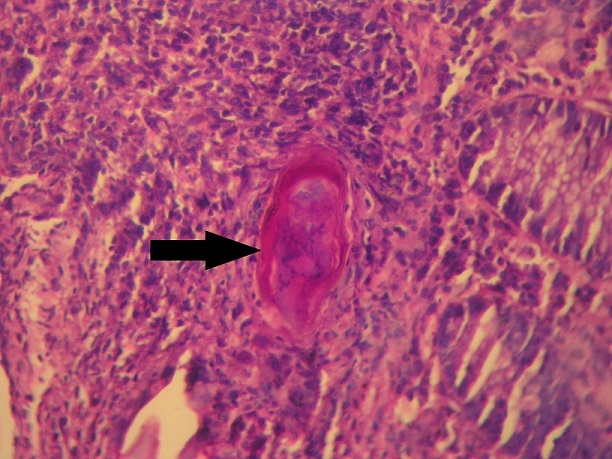
Ovoidal schistosoma egg with terminal spine surrounded by a polymorphous inflammatory infiltrate (black arrow) (hematoxylin and eosin, magnification × 400)

A 20-year-old Tunisian male patient with a past medical history of appendectomy and ileocecal Crohn's disease, presented with abdominal pain and vomiting. He was admitted to the gastroenterology department for clinical check-up. On physical examination, the patient was skinny and pale. Palpation of the left iliac fossa and hypogastrium was painful. He underwent ileocolonoscopy which showed an ulcerated and congested appearance of the upper rectum and sigmoid. Computed tomography scan showed a circumferential thickening of the terminal ileum with luminal stenosis and dilated intestine upstream (Figure 1). Several mesenteric lymphadenopathies were also identified. A fistula between abdominal cavity and skin was disclosed. Stage 2 bilateral sacro-iliitis was objectified on pelvic CT scan. Upper endoscopy was within normal limits. Histological examination of the biopsy specimens of the upper rectum and sigmoid revealed a focally ulcerated colonic epithelium. The lamina propria was fibrous harbouring a polymorphic inflammatory infiltrate including lymphocytes and plasma cells organized in lymphoid follicles admixed with eosinophils and neutrophils. In the submucosa, two well-preserved schistosoma eggs surrounded by a thick shell with a barely visible terminal spine were identified (Figure 2, Figure 3 and Figure 4). The final pathological diagnosis was colonic schistosomiasis associated with Crohn's disease. The patient underwent an ileocecal resection for stenosis of the terminal ileum complicated with enterocutaneous fistula. The postoperative course was uneventful. A stool examination and serology tests were planned for this patient who was lost to follow-up.

## Discussion

Schistosomiasis is an infectious disease, which affects more than 200 million people worldwide. It is endemic in at least three continents namely Africa, Asia, and South America and thus represents a major public health problem of the tropical and sub-tropical areas [3–5]. Of the five species that infect humans, *Schistosoma mansoni*, *Schistosoma haematobium*, and *Schistosoma japonicum* cause the most morbidity [6]. One of the common manifestations of the chronic form of this enteropathogenic disease is intestinal schistosomiasis. The infection is caused by *S. mansoni*, *S. japonicum*, *S. mekongi* and *S. intercalatum* [7]. The reported frequency of intestinal disease in people living in endemic areas infected with *S. mansoni* or *S. japonicum* is usually 10-50% [8]. Intestinal schistosomiasis occurs due to deposition of *Schistosoma* ova in submucosa producing a granulomatous reaction. The inflammatory reaction within the colonic wall depends upon the immune status of the patient, the number of *Schistosoma* eggs and the duration of their staying within the host’s body [9]. Mucosal edema, hemorrhage and ulceration may occur in bowel wall at its early stage, while thickened bowel wall, polyps, or enteric cavity stricture, can be detected at its advanced stage [10]. Most lesions are situated in the large bowel and the rectum as it was the case in our patient whereas small bowel involvement is rare [8]. Intestinal polyposis, ulcers, fistula and strictures have been attributed to *S. mansoni*. In such cases, diffuse, protein-losing enteropathy may occur, with chronic mucohemorrhagic diarrhea, weight loss and anemia [8]. Physical examination of patients with intestinal schistosomiasis reveals a distended abdomen with diffuse or localized tenderness over the transverse and descending colon. In our patient, palpation of the left iliac fossa and hypogastrium was painful. Severe, long-standing granulomatous or polypous lesions may result in partial or complete bowel obstruction and, in rare cases, appendicitis or perforation. Intense dysenteric syndromes are exceptional, but in some cases fatal. The histopathological pattern of enteritis triggered by *Schistosoma mansoni* should be differentiated from other diseases in the course of which epithelioid cell granulomas develop, mostly from Crohn’s disease and tuberculosis, as well as Yersinia infections. Macroscopically, Crohn’s disease is characterized by skip lesions, the involvement of the entire intestinal wall thickness and the presence of ulceration. Nevertheless, in Crohn’s disease, no such thickening and stiffening of colonic wall is noted as it is the case in the small intestine. In addition, in approximately 20% of cases, the rectum shows superficial ulceration and numerous inflammatory pseudopolyps. Similar lesions are encountered in intestinal schistosomiasis, but without the characteristic skip lesions. Macroscopically and radiologically, tuberculous lesions of the colon resemble the lesions seen in schistosomiasis and Crohn’s disease. Yet, usually the former are accompanied by evident mesenteric lymph node enlargement. Lesions resulting from an *Yersinia*infection mostly involve the ultimate segment of the small intestine and the initial segment of the colon, as well as the appendix.

## Conclusion

In view of its clinical similarity to various other diseases, schistosomiasis may induce diagnostic errors. This is why a good collaboration between clinicians and pathologists is crucial, thanks to which clinical information is conveyed that is of great significance in diagnosing such a rare tropical disease. The diagnosis of schistosomiasis lies in detecting *Schistosoma* eggs in the stool, urine or in biopsy material collected from the colon or urinary bladder. Positive serology in itself does not constitute a sufficient foundation for initiating the treatment, but it should prompt a search for live eggs [8, 11]. Previous studies stated that colonoscopic findings are suggestive of schistosomiasis in about 45,3% of patients, but *S mansoni* eggs in feces are detectable in only 11,1% of the patients with colonic biopsies positive for *S mansoni* [12]. Endoscopic findings may not be typical and it is often hard to distinguish *Schistosoma* infection from a chronic inflammatory bowel disease, since these two disorders may show similar patterns. They may also coexist as it was the case in our patient.
